# Outcomes of Laser Conization for Cervical Intraepithelial Neoplasia 2-3 and Microinvasive Cervical Cancer

**DOI:** 10.14740/wjon799w

**Published:** 2014-05-06

**Authors:** Seiji Kanayama, Eriko Nakagawa, Sayaka Ueno, Miho Muraji, Senn Wakahashi, Tamotsu Sudo, Takashi Yamada, Satoshi Yamaguchi, Kiyoshi Fujiwara, Ryuichiro Nishimura

**Affiliations:** a Department of Obstetrics and Gynecology, Nara Medical University, 840 Shijo-cho, Kashihara, Nara 634-8522, Japan; b Department of Gynecologic Oncology, Hyogo Cancer Center, 13-70 Kita-Oji, Akashi, Hyogo 673-8558, Japan

**Keywords:** Conization, Cervical intraepithelial neoplasia, Microinvasive cervical cancer, Laser

## Abstract

**Background:**

Currently, there is no standardized follow-up protocol for patients who undergo laser conization. Therefore, we retrospectively investigated the clinical outcomes of laser conization in patients with high-grade cervical intraepithelial neoplasia 2-3 (CIN 2-3) and microinvasive squamous cell carcinoma and assessed the risks of residual and recurrent lesions of the cervix uteri.

**Methods:**

The medical and pathological records of 91 patients with CIN 2, 580 with CIN 3 and 73 with microinvasive cervical cancer (MIC) who underwent laser conization between January 2000 and December 2011 were retrospectively reviewed.

**Results:**

Positive margins increased with the extent of disease and were observed in 5.5%, 8.9% and 16.4% patients with CIN 2, CIN 3 and MIC, respectively, while residual or recurrent disease was observed in 0%, 3.2% and 13.6% patients, respectively. Examination of specimens obtained through postconization biopsy or hysterectomy revealed that 1.5% and 20% patients with negative and positive margins, respectively, were diagnosed with residual or recurrent lesions. Among patients who were conservatively managed after conization, seven with CIN 3 exhibited residual or recurrent disease, as evidenced by abnormal cytological findings, within 2 years after conization.

**Conclusions:**

Continuous follow-up by cytology and colposcopy, particularly during the first 2 years after conization, can effectively detect early residual or recurrent disease in CIN 3 and MIC patients, regardless of their margin status.

## Introduction

Cervical intraepithelial neoplasia (CIN) is a common disease among sexually active young women. High-grade CIN is considered to be a precursor of invasive cervical cancer and if left untreated, it can be a risk factor for invasive cancer [1]. Conization of the cervix is not only a diagnostic procedure but also the most widely accepted method for treating CIN 3 and microinvasive squamous cell carcinoma [2, 3]. In many of these patients, conization is considered curative for high-grade CIN and microinvasive cervical cancer (MIC), decreasing the risk of invasive cervical cancer by 95% [4].

However, conization may fail, with 5-15% of these patients developing residual or recurrent disease [5-7]. Although follow-up after conization and early detection of treatment failure are important, till date, no standardized follow-up protocol has been developed. Margin involvement, age, menopausal status, human papillomavirus (HPV) persistence and cytological grade are reportedly risk factors for CIN persistence or recurrence after conization [6, 8, 9]. Although the resection margin during conization is considered to be the most important predictor of recurrent disease in patients who undergo cervical conization [6], several studies have reported that CIN persistence or recurrence is independent of margin involvement [8-10].

Therefore, we investigated the clinical outcomes of conization in patients with high-grade CIN and MIC, with particular focus on the status of margin involvement.

## Materials and Methods

We retrospectively investigated the medical and pathological records of 812 patients who underwent cervical conization in the Department of Gynecology at Hyogo Cancer Center Hospital, Japan, between January 2000 and December 2011. The study protocol was approved by the Institutional Review Board of Hyogo Cancer Center Hospital. The indications for conization included persistent (≥ 1 year) CIN 2, CIN 3, or MIC and abnormalities in cytology, biopsy and/or colposcopic findings.

The median age of patients was 37 years (range, 19 - 69 years), with 122 (15.0%) being < 30 years of age and 64 (7.9%) being postmenopausal women. The median follow-up period after conization was 25 months (range, 1 - 128 months). All patients underwent laser conization using a standard technique. Preoperative colposcopy was performed to identify the margins of the lesion. Patients with wide erosive lesions also underwent laser vaporization around the peripheral areas. Surgical specimens were examined by experienced pathologists. Data on the size of each sample, severity of dysplasia, status of endocervical and ectocervical margins or distance from the lesion to the surgical margins and involvement of endocervical glands were recorded.

Margins were considered disease-free when their distance from the CIN lesion was > 1 mm. Condyloma was not considered positive. Positive resection margins were defined as CIN ≥ 2 at the edge of a surgical specimen. After conization, all patients underwent follow-up examinations, including cytological and colposcopic examinations, every 3 months for 2 years. A repeat biopsy was conducted if follow-up colposcopic or cytological findings were abnormal.

Treatment was considered to have failed if residual/recurrent disease, defined as the presence of CIN ≥ 2 after conization biopsy or hysterectomy, was present. Treatment failure within 6 months after conization was considered residual disease, while that at > 6 months after conization was considered recurrent disease.

## Results

Of the 812 patients who underwent conization during the study period, 23 were diagnosed with CIN 1, 91 with CIN 2, 580 with CIN 3, 73 with MIC, 15 with invasive cancer, ten with adenocarcinoma in situ (AIS) and 18 with condyloma or chronic cervicitis (Table 1). We assessed the outcomes for 746 of these patients: 91 with CIN 2, 580 with CIN 3 and 75 with MIC.

**Table 1:** The Patients Included in This Study Were Summarized

	Cases	%
Patients	812	
Age, median (range) years	37	(19 - 69)
19 - 29	122	15.0
30 - 39	383	47.2
40 - 49	243	29.9
50 ≤	64	7.9
Final diagnosis		
CIN 1	23	2.8
CIN 2	91	11.2
CIN 3	580	71.4
Ia 1	71	8.7
Ia 2	2	0.3
Ib 1	15	2.0
Adeno. (AIS/Ia)	12 (10/2)	1.5
Condyloma and others	18	2.2

### CIN 2: follow-up and outcomes

Of the 91 patients with CIN 2, five (5.5%) had positive resection margins. Eighty-nine (97.8%) of these patients underwent conization only and two (2.2%) underwent conization followed by hysterectomy. None of these patients developed residual or recurrent disease.

### CIN 3: follow-up and outcomes

Of the 580 patients with CIN 3, 50 (8.6%) and 530 (91.4%) had positive and negative resection margins, respectively. Sixty-three (10.9%) patients underwent conization followed by immediate hysterectomy, whereas 517 (89.1%) underwent conization followed by conservative management. Ten of the 50 (20%) patients with positive margins and eight of the 530 (1.5%) with negative margins were diagnosed with residual or recurrent disease on the basis of the examination of specimens obtained through postconization biopsy or hysterectomy (Table 2). Of the 50 patients with positive margins, 34 (70%) had ectocervical margin involvement, 12 (24%) had endocervical margin involvement and three (6%) had both. The rates of residual or recurrent lesions in these patients were 11.4%, 33.3% and 66.6%, respectively (Table 3).

**Table 2:** Rates of Residual or Recurrent CIN 3 Disease After Laser Conization Are Shown

	Conizaion + follow-up	Conization + hysterectomy	Total
Negative margin % (n)	0.6 (3/476)	5.5 (5/54)	1.5 (8/530)
Positive margin % (n)	9.7 (4/41)	66.7 (6/9)	20 (10/50)

**Table 3:** Sites of Margin Involvement and Rates of Residual or Recurrent Disease Are Shown

Site of margin involvement	Residual/recurrent	%
Ectocervix	4/35	11.4
Endocervix	4/12	33.3
Bilateral	2/3	66.6

Of the 517 patients who were conservatively managed after conization, seven (1.3%) had residual or recurrent disease (Table 4). During post-treatment follow-up, all seven of these patients presented with abnormal cytological findings. Specifically, treatment failure was represented by CIN 2 in one patient, CIN 3 in four patients, AIS in one patient and invasive squamous cell carcinoma in one patient. One patient underwent photodynamic therapy (PDT); however, CIN persisted and she subsequently underwent repeat conization. Two patients underwent repeat conization; both remained disease-free. Of the four patients who underwent hysterectomy, one presented with invasive cancer and underwent adjuvant whole pelvic irradiation after surgery.

**Table 4:** Summary of Residual or Recurrent Lesions After Conization and Conservative Follow-Up Is Shown

	CIN grade	Margin	Time to presentation of abnormal cytology	Time to retreatment	Retreatment method	Residual lesion
1	CIN 3	positive	3 M	10 M	ATH	CIN 3
2	CIN 3	negative	5 M	16 M	ATH	AIS
3	CIN 3	positive	13 M	17 M	ATH	CIN 3
4	CIN 3	positive	4 M	9 M	re-cone	CIN 2
5	CIN 3	negative	3 M	22 M	PDT→ re-cone	CIN 2
6	CIN 3	negative	3 M	14 M	re-cone → 12 M ATH	CIN 3
7	CIN 3	positive	10 M	28 M	ATH→ pTIb1 CCRT	Invasive
					LVSI+, pelvic LN+	SCC

TAH: total abdominal hysterectomy; PDT: photodynamic therapy; LVSI: lymph vascular space involvement; CCRT: concurrent chemo-radiation therapy.

### MIC: follow-up and outcomes

We also reviewed 73 patients with MIC: 71 with stage Ia1 and 2 with stage Ia2 disease. All patients were retrospectively staged according to the International Federation of Gynecology and Obstetrics (FIGO) guidelines of 1994. Of these 73 patients, 24 (32.8%) underwent conization followed by conservative management and 49 (67.2%) underwent conization followed by immediate hysterectomy. Lymph-vascular space involvement (LVSI) was observed in three patients with stage Ia1 and one with stage Ia2 disease. Two patients with stage Ia2 disease underwent conization followed immediately by radical hysterectomy, one of whom had a residual lesion in the ectocervix. One of 24 (4.2%) patients treated by conization only had a positive resection margin, although she was treated successfully.

Of 72 patients who were conservatively managed after conization, none showed residual or recurrent disease. In contrast, of the 49 patients treated by conization followed by hysterectomy, nine (18.4%) had residual lesions, including four of 38 patients (10.5%) with negative margins (two with CIN 3 and two with MIC) and five of 11 patients (45.4%) with positive margins (one with CIN 3 and four with MIC).

We found that 5.5% patients with CIN 2, 8.9% with CIN 3 and 16.4% with MIC had positive margins; the rate increased with the extent of disease. Similarly, 0% patients with CIN 2, 3.2% with CIN 3 and 13.6% with MIC developed residual or recurrent disease (Table 5).

**Table 5:** Rates of Margin Involvement and Residual or Recurrent Disease Are Shown

	Margin involvement (%)	Residual/recurrent (%)
CIN 2	5.5	0.0
CIN 3	8.9	3.2
MIC	16.4	13.6

## Discussion

In general, the overall surgical success rate of conization for CIN is > 90% [5, 6]. Most treatment failures are caused by CIN persistence or relapse and almost all these treatment failures are diagnosed within 2 years of conization. Although there is a need for careful follow-up and early detection of treatment failure, till date, no standardized follow-up protocol has been developed. Follow-up procedures may include cytology, endocervical biopsy, HPV DNA testing and/or colposcopy [7, 8]. Conventional follow-up after conization usually includes periodic cytological testing and colposcopy. We found that, of 517 patients with CIN 3 who were conservatively managed after conization, only seven (1.3%) developed residual or recurrent disease. All seven of these patients had a cytological abnormality, suggesting the value of follow-up cytology examinations. In contrast, several studies reported relatively high false-negative rates with follow-up cytology [9]. Factors such as margin involvement, age, menopausal status and HPV persistence are reportedly risk factors for CIN persistence or recurrence after conization [6, 10, 11].

Among these risk factors, the presence of CIN in the conization margin is strongly associated with treatment failure [6]. For example, a study that examined the outcomes of large-loop excision of the transformation zone in 225 patients with CIN 3 found that the incidence of CIN was 16.5% after incomplete excision and 1.9% after complete excision [12]. Another study reported results [13] similar to ours, documenting that ten of 50 (20%) and eight of 530 (1.5%) patients with positive and negative resection margins were diagnosed with residual or recurrent lesions on the basis of the examination of specimens obtained through postconization biopsy or hysterectomy. A meta-analysis of studies published between 1960 and 2007 showed that the relative risk of residual or recurrent high-grade CIN after conization was 6.09 (95% confidence interval (CI), 3.87 - 9.60) in patients with positive relative to negative margins [14].

Residual or recurrent lesions have been reported in patients with negative margins [15, 16]. We found that 1.5% of our patients with negative margins had residual or recurrent disease, suggesting that, even after complete resection, the risk of residual or recurrent disease is not negligible. Therefore, it is necessary to identify other factors predictive of residual or recurrent disease, which is a major concern associated with conservative treatment for CIN and MIC of the uterine cervix.

HPV testing may also predict treatment failure after conization [17-19]. For example, a study that included HPV DNA testing before and after the loop electrosurgical excision procedure (LEEP) for patients with high-grade CIN found that the persistence of high-risk HPV DNA in 30.6% patients with positive surgical margins was correlated with HPV DNA positivity [19]. A review of 11 studies showed that the negative predictive value of high-risk HPV testing was 83% [20]. HPV testing may detect residual or recurrent CIN more quickly than follow-up cytology, with a higher sensitivity and a similar specificity. However, it is not routinely performed during follow-up after conization. However, because the persistence of HPV infection after conization is a high-risk factor, these tests may be able to predict residual or recurrent disease.

The duration of post-conization follow-up for patients with high-grade CIN or MIC has not been standardized worldwide. Residual or recurrent disease usually occurs within two years after conservative treatment [19, 21], which is consistent with our findings. The guidelines in several countries advocate that women should be followed-up by cytology examination for at least two years. However, several cases of late recurrence over a period of 20 years have been reported [14, 22]. The cumulative risk of invasion after 8 years was reportedly 5.8 per 1,000 patients or five times greater than that in the general population [22]. These results indicate the need for continuous and longer follow-up durations for some patients, in addition to individualization of follow-up periods on the basis of each patient’s risk factors for recurrence. Positivity for high-risk HPV at 3 to 6 months after conization is reportedly a risk factor for persistent or recurrent cytological and pathological abnormalities [4, 5]. This suggests that patients who are positive for high-risk HPV should undergo frequent and meticulous post-therapy surveillance, whereas those with negative results do not require such high-level surveillance and can undergo routine follow-up [23]. In conclusion, our results confirmed the efficacy of continuous follow-up by cytology and colposcopy, particularly during the 2 years after conization, even for women with negative resection margins. The duration of follow-up is not yet determined, although several studies recommend that patients should be followed-up for more than 10 years. Excessive surveillance, particularly for low-risk patients, can be avoided by identifying more reliable risk factors that can predict the likelihood of residual or recurrent disease.
